# The algorithmic self: reimagining conscious leadership in posthuman education: toward a posthuman ethics of educational awareness

**DOI:** 10.3389/fpsyg.2026.1741093

**Published:** 2026-03-26

**Authors:** Okyanus Işık Seda Yılmaz

**Affiliations:** Postdoctoral Researcher (Independent), Eskişehir, Türkiye

**Keywords:** AI-mediated governance, algorithmic self, conscious leadership, posthuman education, relational ethics

## Abstract

The integration of artificial intelligence into educational systems is reshaping how leadership, decision-making, and institutional accountability are enacted. While existing research on AI and educational leadership often focuses on governance, efficiency, and data-driven management, less attention has been given to the ontological and ethical implications of algorithmic mediation. This paper addresses this gap by developing a conceptual model of conscious leadership grounded in posthuman philosophy, transpersonal psychology, and relational leadership theory. Using a conceptual synthesis methodology, the study introduces the notion of the algorithmic self to describe a relational configuration of awareness emerging at the intersection of human cognition and technological mediation. The proposed model integrates psychological depth, ethical reflexivity, and technological attunement as interdependent dimensions of leadership practice within AI-mediated environments. Rather than positioning technology as either a threat or a solution, the framework situates leadership within hybrid human–machine systems where agency and responsibility are dynamically negotiated. The article contributes to theoretical discussions in educational leadership and philosophical psychology by reframing leadership as a relational and ethically mediated process shaped by socio-technical infrastructures. It further connects posthuman ethics with evolutionary cognition, suggesting that leadership in the algorithmic age involves both ethical reflection and cognitive adaptation within complex digital ecologies.

## 1 Introduction

The accelerating integration of artificial intelligence into educational systems has prompted renewed reflection on the nature of leadership, agency, and responsibility in contemporary schooling. Digital platforms, predictive analytics, and algorithmic decision-support systems increasingly mediate assessment, resource allocation, communication, and governance (Knox, 2023; Selwyn, 2019; Williamson, 2017). International policy frameworks have increasingly recognized that AI systems are reshaping educational governance structures, calling for transparency, accountability, and meaningful human oversight in institutional decision-making (UNESCO, 2021; OECD, 2021). While these developments are often framed in terms of efficiency and innovation, they also raise deeper questions concerning how authority, judgment, and ethical responsibility are constituted within hybrid human–technological environments. In this context, educational leadership can no longer be understood solely as an interpersonal or organizational function; it unfolds within socio-technical ecologies that shape perception, decision-making, and accountability.

Since 2023, the rapid diffusion of generative AI in organizational work has made these governance questions more operational and time-sensitive for educational leadership, because leaders are now using GenAI for concrete managerial and instructional tasks (e.g., planning, documentation, communication, and evaluation workflows), thereby shifting what counts as “decision support” in day-to-day leadership practice (Berkovich and Benoliel, 2025). In parallel, global guidance has emphasized that institutions should validate GenAI use for ethical and pedagogical appropriateness, and should strengthen human agency, accountability, and data protection in educational settings (UNESCO, 2023).

Educational leadership scholarship has, over recent decades, moved beyond purely managerial paradigms toward relational, distributed, and value-based models (Uhl-Bien, 2006; Day, 2014). These approaches have significantly decentered hierarchical control and emphasized shared meaning-making. Nevertheless, even within such relational frameworks, leadership is typically anchored in human intentionality as the primary locus of agency. This observation does not deny the diversity of leadership paradigms across cultural and theoretical traditions. Rather, it highlights a recurring analytical tendency within much of the literature to retain the human subject as the ultimate locus of agency, even when leadership is conceptualized as relational or distributed. The present argument therefore seeks to extend—rather than dismiss—existing frameworks by examining how algorithmic mediation subtly reconfigures the ontological assumptions underlying agency and responsibility. As algorithmic systems increasingly participate in institutional processes, this human-centered assumption warrants reconsideration. Rather than displacing human leadership, AI-mediated infrastructures reconfigure the conditions under which agency and responsibility are enacted.

Recent educational leadership scholarship has begun to address this reconfiguration more directly by examining AI as a participant in decision-making and governance—raising practical concerns about accountability, interpretability, and the redistribution of responsibility when algorithmic recommendations enter leadership judgments (Wang, 2021, 2024). At the policy level, the European Union’s Artificial Intelligence Act has further intensified the accountability agenda by establishing harmonized rules and risk-based obligations for AI systems, reinforcing expectations around governance, transparency, and oversight that will shape public-sector and education-adjacent deployments (European Parliament and Council of the European Union, 2024).

Current discussions of AI-driven educational leadership frequently adopt an instrumental orientation, focusing on data optimization, performance metrics, and governance efficiency (Knox, 2023; Jandrić and Ford, 2020). While such analyses are indispensable, they often leave underexamined the phenomenological and ethical dimensions of awareness itself—how consciousness, rather than cognition alone, participates in shaping leadership within algorithmically mediated systems. The present study addresses this gap by foregrounding consciousness as both the subject and medium of leadership in posthuman education.

In addition, the expansion of algorithmic decision infrastructures in education governance has sharpened concerns about bias, discrimination, and socio-technical inequalities—issues that extend beyond “efficiency” and require leaders to interpret algorithmic outputs within normative and contextual frames rather than treating them as neutral evidence (Eynon, 2023; Wang, 2024). In this study, posthumanism is understood not as the replacement of the human, but as an ontological shift where agency is distributed across hybrid human-technological networks.

The term posthuman is used here not to suggest the obsolescence of the human subject, but to denote a shift in ontological perspective: human agency is understood as relationally constituted within networks of technological, ecological, and social interdependence (Braidotti, 2013; Ferrando, 2019). Within this framework, leadership is reconceived as an emergent practice unfolding across distributed systems of awareness. Building on transpersonal psychology (Ferrer, 2002; Wilber, 2000) and phenomenology, this paper develops the concept of the algorithmic self—a relational configuration of consciousness arising at the intersection of human intuition and algorithmic mediation. The algorithmic self does not replace the human subject; rather, it describes how awareness is extended, scaffolded, and reshaped within AI-mediated environments.

Contemporary international guidance on AI in education underscores the necessity of maintaining human-centered governance models in algorithmically mediated environments, particularly in light of emerging regulatory frameworks such as the European Union AI Act (European Parliament and Council of the European Union, 2024; UNESCO, 2023). Accordingly, this study asks: How can educational leadership be reimagined as a consciousness-based, ethically reflexive practice within posthuman systems mediated by artificial intelligence? To address this question, the paper advances a conceptual model of conscious leadership integrating psychological depth, ethical reflexivity, and technological attunement as interdependent dimensions of awareness in hybrid human–machine ecologies.

To strengthen the governance relevance of this framing, the model is also aligned with the recent shift toward “algorithmic decisions” in education governance, where AI-informed processes influence institutional choices and accountability logics—thereby making leadership responsibility more distributed but not less demanding in terms of ethical judgment (Wang, 2024).

In addition to posthuman and transpersonal perspectives, the algorithmic self can be interpreted through evolutionary cognition. Human consciousness evolved to navigate social complexity, detect agency, and sustain cooperation within groups (Barkow et al., 1992; Buss, 2019). In AI-mediated contexts, these evolved mechanisms remain operative, often producing anthropomorphic responses to digital systems (Waytz et al., 2010). The algorithmic self thus reflects both evolutionary inheritance and technological transformation: a culturally scaffolded extension of adaptive cognition (Clark and Chalmers, 1998) within contemporary educational infrastructures.

By situating leadership as a form of relational consciousness, this study contributes to theoretical and philosophical psychology—particularly debates concerning awareness, agency, and moral cognition in human–machine interaction—while offering an integrative framework for rethinking educational leadership in the algorithmic age.

### 1.1 Theoretical context: posthuman consciousness and leadership

The emergence of posthuman and transpersonal paradigms invites a critical re-examination of assumptions embedded within educational leadership theory. While contemporary scholarship has increasingly emphasized relational, distributed, and value-based models of leadership (Uhl-Bien, 2006; Day, 2014), much of the field continues to presuppose a primarily human-centered locus of agency, where leadership ultimately resides in the intentionality and rationality of human actors. It should be noted, however, that this characterization applies unevenly across national and cultural contexts. In several educational traditions—most notably those of Scandinavian countries—leadership ideals have long emphasized egalitarianism, relational responsibility, and value-based decision-making (Møller and Skedsmo, 2013; Møller, 2017).

The present critique is therefore directed not at educational leadership as a whole, but at the dominant theoretical frameworks that have anchored agency primarily within human intentionality, even within relational models. Even relational frameworks, though decentering hierarchical control, typically retain the human subject as the principal source of meaning and responsibility. In this sense, leadership theory has largely operated within anthropocentric ontological boundaries. In response, contemporary scholarship suggests a “post-anthropocentric” shift that recognizes the entanglements of human and non-human actors in the co-production of educational spaces (Bayne et al., 2020; Gourlay, 2020).

Recent work in digital education and postdigital theory has deepened this critique by examining how platforms, data infrastructures, and algorithmic systems participate in shaping institutional practices and epistemic authority, rather than merely supporting human actors (Jandrić et al., 2018; Williamson et al., 2023). Such analyses reinforce the argument that educational spaces are co-produced within socio-technical assemblages, thereby challenging strictly anthropocentric accounts of leadership and agency.

Posthuman philosophy complicates this assumption. As articulated by Braidotti (2013) and Ferrando (2019), posthumanism does not negate human agency but situates it within networks of technological, ecological, and material co-agency. Rather than envisioning the dissolution of the human subject, posthuman thought reframes subjectivity as relational, embodied, and distributed. Within this shift, leadership can be understood less as a possession of an individual actor and more as an emergent property of socio-technical assemblages—networks in which human intention, algorithmic processes, and institutional infrastructures interact dynamically.

Research on the datafication of education has demonstrated how digital infrastructures and platform-based systems participate in shaping institutional decision-making and accountability processes, thereby reconfiguring governance logics within contemporary educational systems (Williamson and Eynon, 2020; Williamson, 2021; Williamson et al., 2023).

In educational research specifically, the increasing role of datafication and algorithmic governance has further supported this relational view of agency, demonstrating how decision-making authority may be reconfigured through automated classification, prediction, and evaluation systems (Eynon, 2023; Wang, 2024). These developments do not eliminate human leadership but situate it within algorithmically mediated environments that shape what can be perceived, prioritized, and acted upon.

From a transpersonal psychological perspective, consciousness likewise exceeds the boundaries of the egoic self. Ferrer (2002) and Wilber (2000) describe awareness as participatory and relational, arising through interaction rather than isolation. Applied to educational contexts, this view reframes leadership as awareness-in-action: a reflective attunement to psychological depth, ethical responsibility, and systemic interdependence. Leadership, in this framework, is not abandoned as a human capacity, but expanded—grounded in inner coherence while responsive to broader relational fields.

Contemporary research on distributed and extended cognition has renewed interest in how cognitive processes are scaffolded by digital environments, suggesting that cognitive activity can be distributed across human–technological systems rather than confined to isolated individuals (Heersmink, 2022; Risko and Gilbert, 2016; Wang, 2024). While not explicitly posthuman in orientation, this body of work empirically supports the claim that agency and cognition are environmentally embedded and technologically mediated.

In the age of artificial intelligence, these theoretical orientations acquire renewed relevance. Algorithmic systems increasingly participate in educational decision-making, shaping assessment, resource allocation, and communication. Such mediation does not eliminate human leadership; rather, it transforms the conditions under which leadership is enacted. Authority becomes negotiated within hybrid human–machine ecologies, where cognition and judgment are distributed across biological and computational processes. This shift calls for what may be described as posthuman reflexivity: an awareness of how one’s perception, decision-making, and ethical stance are co-constituted by technological infrastructures.

Recent studies of algorithmic decision-making in educational governance further demonstrate that leaders must interpret, contextualize, and sometimes contest algorithmic outputs, rather than treating them as neutral or self-evident forms of authority (Wang, 2024). This empirical strand reinforces the need for reflexive awareness in environments where computational systems influence institutional judgments.

More recent psychological research on human–AI interaction has shown that individuals systematically attribute agency, intentionality, and even moral standing to algorithmic systems under certain conditions, thereby confirming that evolved social-cognitive mechanisms remain operative in digital contexts (Glikson and Woolley, 2020; Longoni et al., 2019). Although these studies predate the generative AI surge, they provide empirical grounding for the claim that anthropomorphic and trust-related dynamics persist in algorithmically mediated environments.

From an evolutionary-cognitive perspective, this transformation can be interpreted as an extension rather than a rupture of human adaptive architecture. The capacities for cooperation, agency detection, and moral reasoning evolved under pressures of social complexity (Buss, 2019; Tomasello, 2019). In algorithmically mediated environments, these adaptive mechanisms remain active, often producing anthropomorphic responses to digital systems (Waytz et al., 2010). The algorithmic self, therefore, should not be understood as a replacement of the human subject, but as a culturally scaffolded configuration of consciousness (Clark and Chalmers, 1998)—a relational mode of awareness emerging at the intersection of evolved cognition and technological mediation.

Within this conceptual space, leadership becomes neither purely human nor purely technological, but an ethically mediated practice unfolding within distributed systems of awareness. The challenge is not to displace the human, but to cultivate forms of consciousness capable of engaging algorithmic environments reflectively and responsibly.

## 2 Conceptual framework: the conscious leadership model in posthuman education

The Conscious Leadership Model proposed in this paper conceptualizes leadership as a dynamic field of awareness emerging at the intersection of human consciousness, technological mediation, and ethical responsibility. Contemporary educational leadership research has significantly expanded beyond purely managerial paradigms to include relational, distributed, transformational, and complexity-oriented perspectives (Uhl-Bien, 2006; Day, 2014). These approaches have emphasized shared meaning-making, adaptive capacity, and systemic interdependence within organizations. Yet, as digital infrastructures and AI systems increasingly mediate institutional processes, even these relational frameworks encounter new ontological questions regarding how agency, responsibility, and awareness are constituted within hybrid human–technological ecologies.

In an era marked by digital transformation, ecological uncertainty, and moral pluralism, leadership cannot be reduced to behavioral technique or procedural coordination alone. Rather, it increasingly unfolds within socio-technical assemblages that shape perception, judgment, and institutional culture (Knox, 2023; Selwyn, 2021; Williamson, 2017). The Conscious Leadership Model responds to this emerging context by reframing leadership as an ontological and ethical practice—a cultivation of awareness integrating psychological depth, ethical reflexivity, and technological attunement. Instead of opposing existing leadership theories, the model extends them, situating relational and distributed leadership within a broader ecology of consciousness responsive to posthuman conditions.

This framework draws on three converging streams of thought. First, depth and transpersonal psychology (Jung, 1959; Ferrer, 2002; Wilber, 2000) provide an account of consciousness as layered, evolving, and relational, offering the inner foundation for reflective leadership. Second, philosophical posthumanism (Braidotti, 2013; Ferrando, 2019; Nayar, 2014) challenges exclusively human-centered epistemologies and situates agency within networks of human and non-human co-constitution. Third, ethics of technology (Floridi, 2014; Verbeek, 2011) illuminate how digital systems mediate moral agency and institutional power, calling for a renewed form of ethical awareness among leaders. By synthesizing these traditions, the model positions leadership not as the property of a sovereign individual, but as a relational capacity emerging within complex socio-technical systems.

Within this integrative view, leadership remains humanly grounded yet posthumanly adaptive. Psychological depth anchors emotional and moral coherence; ethical reflexivity guides value-oriented decision-making amid data-driven complexity; and technological attunement cultivates reflective engagement with algorithmic infrastructures. Together, these dimensions form an ecology of awareness—an understanding of leadership as both a personal and systemic capacity shaped by distributed forms of cognition and responsibility.

### 2.1 Psychological depth: Inner awareness in complex systems

Psychological depth refers to the leader’s capacity for sustained self-reflection, emotional integration, and empathic presence within complex and evolving educational environments. Drawing on depth and transpersonal psychology (Jung, 1959; Ferrer, 2002; Wilber, 2000), this dimension frames leadership as an inner process of consciousness development rather than merely an external coordination of roles or tasks. Jung’s (1959) notion of individuation—integrating the conscious and unconscious dimensions of the self—provides a foundation for understanding how authentic leadership emerges through self-knowledge and the reconciliation of internal tensions. Similarly, Ferrer’s (2002) participatory model situates consciousness within relational and embodied contexts, emphasizing interconnection as a basis for ethical responsiveness.

While relational and distributed leadership theories highlight shared meaning-making and collective agency (Uhl-Bien, 2006; Day, 2014), psychological depth underscores the interior conditions that make such relational processes sustainable. Without reflective self-awareness and emotional integration, distributed leadership risks becoming procedural rather than genuinely participatory. In this sense, psychological depth complements contemporary leadership scholarship by grounding systemic collaboration in inner coherence.

Within posthuman educational systems—where information, affect, and decision-making circulate through hybrid human–technological networks—psychological depth becomes a stabilizing ethical force. The leader’s inner coherence enables discernment amid algorithmic acceleration, data saturation, and institutional complexity. Emotional intelligence (Goleman, 1998) and systems awareness (Senge, 1990) thus converge with transpersonal insight to form a multidimensional consciousness—one that perceives education as both a psychological and moral ecosystem shaped by socio-technical mediation.

Cultivating psychological depth therefore means nurturing the interior capacities that allow leaders to respond to complexity without fragmentation. As leadership theorists argue (Day, 2014; Kets de Vries, 2006), self-awareness and emotional maturity are not private virtues but public capacities shaping institutional culture. In AI-mediated environments, where algorithmic outputs may appear authoritative and emotionally neutral, the conscious leader integrates emotion, intuition, and reflection as sources of ethical wisdom—ensuring that technological efficiency does not eclipse moral discernment.

### 2.2 Ethical reflexivity: navigating moral ambiguity in hybrid systems

As algorithmic and data-driven systems increasingly shape educational governance and institutional decision-making, ethical reflexivity emerges as a central dimension of conscious leadership. This dimension concerns the leader’s capacity to discern, negotiate, and act within morally complex contexts in which responsibility is mediated across human actors, technological systems, and institutional infrastructures. The posthuman condition—described by Braidotti (2013) as a decentering of the human—does not eliminate agency but complicates traditional assumptions about accountability, authorship, and control. In AI-mediated environments, ethical responsibility becomes relational and distributed, requiring leaders to cultivate reflexive awareness of how decisions are shaped by socio-technical arrangements.

Recent empirical research on human–AI decision-making further supports this relational account of responsibility. Studies have shown that individuals often recalibrate trust in algorithmic systems depending on perceived transparency, explainability, and fairness, and that responsibility attribution becomes diffuse when automated systems are involved in consequential decisions (Glikson and Woolley, 2020; Longoni et al., 2019; Dignum, 2019). In governance contexts, this diffusion does not eliminate human accountability but reshapes how leaders interpret, justify, and assume responsibility for algorithmically mediated outcomes. Such findings reinforce the need for ethical reflexivity as a conscious capacity to interrogate the socio-technical conditions under which authority is exercised.

Recent scholarship on algorithmic bias and automated decision systems in education further underscores the ethical stakes of leadership in AI-mediated environments. Research has demonstrated that predictive analytics and automated classification systems can reproduce or amplify existing structural inequalities, particularly when training data encode historical disparities (Holmes et al., 2022; Eynon, 2023). In such contexts, leaders are not merely users of technical systems but interpreters of algorithmic outputs whose ethical responsibility includes questioning underlying data assumptions, evaluating potential bias, and safeguarding procedural justice. Empirical studies on algorithmic accountability further indicate that when decision-support systems are perceived as opaque, individuals may either over-rely on or unduly distrust automated recommendations, complicating the attribution of responsibility (Glikson and Woolley, 2020). These dynamics reinforce the necessity of ethical reflexivity as an ongoing, institutionally embedded practice rather than a purely individual moral stance.

Drawing on the relational ethics of Gilligan (1982) and the dialogical philosophy of Levinas (1969), ethical reflexivity can be understood as sustained attentiveness to the Other—human, digital, and ecological. Rather than reducing ethics to procedural compliance or rule-based governance, this perspective frames moral action as responsiveness within interdependent systems. Bauman’s (1993) account of moral ambivalence in late modernity further underscores that ethical responsibility cannot be secured solely through policy frameworks but must be enacted through reflective judgment. Within educational leadership, this involves recognizing how data infrastructures encode values, how algorithmic systems may amplify structural inequalities (Holmes et al., 2022; Zuboff, 2019), and how digital mediation shapes what is recognized as fairness, merit, or inclusion. This reflexivity is not merely a personal choice but a systemic necessity, as emerging regulatory frameworks like the EU AI Act (2024) demand a move toward “human-in-the-loop” governance and algorithmic accountability in public sectors (Dignum, 2019; Floridi and Cowls, 2019).

This perspective resonates with scholarship on ethical and responsible leadership, which emphasizes moral attentiveness, relational accountability, and value-based decision-making within complex organizations (Day, 2014; Uhl-Bien, 2006). In hybrid human–algorithmic systems, ethical reflexivity extends these traditions by incorporating technological mediation into the field of moral consideration. Floridi’s (2014) concept of the “infosphere” further clarifies that digital environments constitute moral ecosystems in which human intentions and algorithmic operations co-produce outcomes. Leaders operating within such ecosystems are increasingly required to move beyond compliance-driven ethics toward dialogical and relational forms of responsibility grounded in transparency, care, and critical awareness.

Ethical reflexivity, in this sense, is both cognitive and affective: the capacity to recognize moral tension, interrogate normative assumptions, and respond with discernment rather than reactivity. By cultivating such awareness, educational leaders sustain the moral imagination necessary to navigate algorithmic authority while preserving justice, empathy, and institutional trust in technologically mediated contexts.

### 2.3 Technological attunement: integrating human and algorithmic intelligences

Technological attunement refers to the leader’s capacity to engage reflectively with the digital infrastructures that mediate communication, learning, and decision-making in contemporary educational environments. Recent research on AI-assisted decision-making and human–AI collaboration has further clarified how algorithmic systems influence cognitive delegation, judgment calibration, and institutional reasoning processes. Studies indicate that when algorithmic recommendations are integrated into professional contexts, human actors tend to adjust their confidence, override thresholds, and responsibility perceptions depending on system transparency and perceived competence (Glikson and Woolley, 2020; Logg et al., 2019; Castelo et al., 2019). Within educational settings, this suggests that technological attunement involves not only technical literacy but also meta-cognitive awareness of when and how to rely on algorithmic outputs. Rather than replacing human judgment, AI systems participate in shaping institutional cognition, requiring leaders to continuously interpret, contextualize, and critically evaluate automated recommendations. As schools and universities increasingly operate through algorithmic governance, data analytics, and AI-assisted systems (Williamson, 2017; Knox, 2023), leadership is enacted within socio-technical contexts that shape perception, coordination, and institutional accountability. Rather than displacing human agency, these developments reconfigure the conditions under which agency is exercised.

The notion of the extended mind (Clark and Chalmers, 1998) provides a useful epistemic lens for understanding this transformation. It suggests that cognitive processes can be scaffolded and distributed across tools, artifacts, and environments. Applied cautiously to educational leadership, this perspective highlights how decision-making and judgment are increasingly mediated by algorithmic systems without implying the dissolution of human responsibility. Technological attunement, in this sense, involves awareness of how digital infrastructures participate in shaping institutional cognition.

This orientation resonates with Haraway’s (1991) metaphor of the cyborg as a figure of hybrid relationality—an analytic device for thinking through human–machine entanglements rather than a literal erasure of human distinctiveness. Leaders attuned to technology recognize that digital tools are not neutral instruments; they configure perception, emotion, and institutional behavior. Verbeek (2011) similarly argues that technologies mediate moral experience by influencing how situations are interpreted and acted upon. From this standpoint, technological attunement requires cultivating reflexive awareness of how algorithms and platforms embody particular epistemologies, values, and power relations. As leadership becomes increasingly entangled with opaque computational processes, the capacity to lead requires “learning to work with the black box”—not by achieving total technical transparency, but by developing a pedagogical and relational stance toward AI’s outputs (Bearman and Ajjawi, 2023). This involves recognizing that technological tools are not neutral instruments but active participants that reconfigure institutional cognition.

Leadership scholarship increasingly acknowledges the importance of digital competence and adaptive capacity in complex environments (Day, 2014; Uhl-Bien, 2006). Technological attunement extends these discussions by foregrounding ethical and phenomenological sensitivity to technological mediation. Rather than framing technology as either a threat or a solution, this dimension emphasizes reflective engagement—ensuring that algorithmic efficiency does not eclipse moral discernment and relational accountability.

From an educational perspective, technological attunement involves translating critical digital literacy into institutional wisdom. Recent scholarship in educational leadership and digital governance has begun to examine how artificial intelligence reshapes institutional authority, accountability, and professional judgment within schools and higher education. Studies on data-driven and AI-mediated leadership practices highlight that algorithmic systems increasingly inform performance evaluation, student monitoring, and resource allocation, thereby reconfiguring how leaders interpret evidence and exercise discretion (Knox, 2023; Selwyn, 2021; Williamson et al., 2023). Rather than positioning leaders as passive recipients of technical outputs, this body of work frames leadership as an interpretive practice in which algorithmic classifications and predictive analytics must be critically contextualized within institutional values and equity commitments. In this sense, technological attunement involves cultivating an informed awareness of how digital infrastructures shape what counts as knowledge, risk, and institutional success in contemporary education systems. It includes recognizing how data infrastructures shape inclusion and exclusion (Williamson, 2017), interrogating the normative assumptions embedded in algorithmic systems, and fostering cultures of mindful use rather than passive dependence. When guided by awareness, technology can extend institutional capacities for coordination and insight; when engaged uncritically, it risks amplifying bias and alienation.

Within this framework, the algorithmic self designates a relational configuration of consciousness operating at the intersection of evolved social cognition and digital mediation. Drawing on Clark and Chalmers’s (1998) notion of cognitive scaffolding, it captures how human awareness is extended through technological systems while remaining ethically accountable. The conscious leader thus embodies technological attunement not as technological mastery, but as reflective participation—remaining anchored in human values while navigating algorithmic complexity.

### 2.4 Integrative view: consciousness as relational field

The three dimensions outlined above converge in what this study conceptualizes as conscious presence—an integrative mode of awareness through which psychological depth, ethical reflexivity, and technological attunement dynamically interact. Within this framework, consciousness is approached not solely as an internal attribute of the individual, but as a relational field emerging through interaction with human, technological, and ecological systems (Varela et al., 1991). Such a perspective does not deny individual subjectivity; rather, it situates it within processes of co-constitution and embodied engagement.

Drawing on systems thinking (Senge, 1990) and enactive cognition (Varela et al., 1991), this integrative view reframes educational leadership as a living ecology of awareness. Recent work in postdigital education and sociomaterial theory further supports this relational framing by emphasizing that educational practices are enacted through entanglements of human actors, digital platforms, material infrastructures, and policy architectures (Jandrić et al., 2018; Gourlay, 2020; Bayne et al., 2020). Rather than treating technology as an external tool, postdigital scholarship conceptualizes education as constituted within hybrid assemblages where epistemic authority, institutional norms, and professional identities are co-produced. From this perspective, leadership cannot be understood as an exclusively human attribute but must be interpreted as emerging within sociotechnical relations that shape perception, legitimacy, and action. This ontological shift strengthens the argument that consciousness, in educational leadership, operates as a relational field rather than a bounded psychological property. Psychological depth anchors the leader in reflective self-knowledge; ethical reflexivity sustains moral responsiveness within complex contexts; and technological attunement extends perception and judgment across socio-technical networks. In dialog with relational and complexity-oriented leadership theories (Uhl-Bien, 2006; Day, 2014), this model suggests that leadership can be reconceptualized as a process of attunement rather than control—a continuous negotiation between self and system, awareness and action.

When these dimensions are aligned, they create conditions for what Ferrer (2002) describes as participatory knowing: a mode of awareness that recognizes knowledge, agency, and responsibility as co-constructed within relational fields. From this standpoint, leadership is neither reduced to hierarchical authority nor dissolved into abstract networks; instead, it becomes an ethically mediated practice unfolding within distributed systems of cognition.

In educational contexts shaped by artificial intelligence and digital governance, such an integrative orientation invites leaders to cultivate collective capacities for reflection, dialog, and ethical co-creation. Rather than replacing established leadership models, this perspective extends them—foregrounding consciousness as a dimension through which relational, distributed, and adaptive leadership practices gain ontological depth. Within posthuman education, consciousness thus functions not as a mystical abstraction, but as a practical orientation toward awareness, responsibility, and systemic interdependence.

**FIGURE 1:**
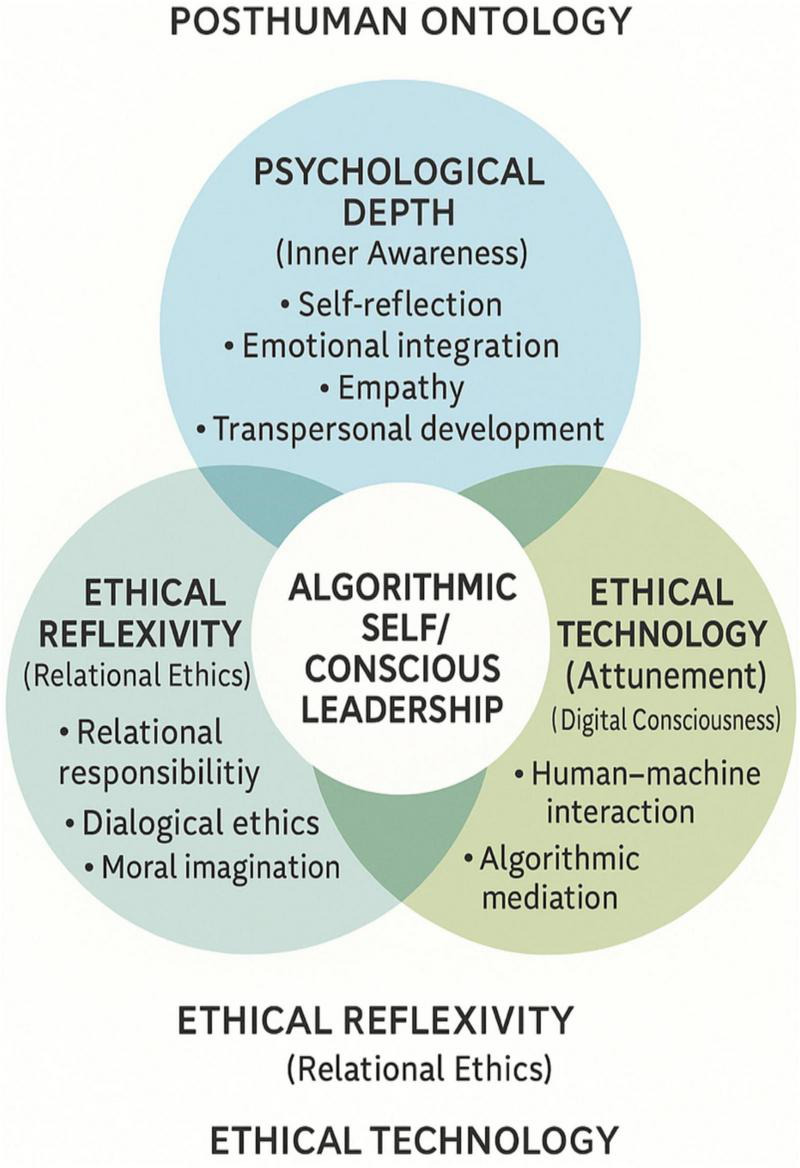
The conscious leadership model in posthuman education.

These interrelated dimensions and their philosophical foundations are summarized in Figure 1.

Figure 1 illustrates the Conscious Leadership Model in Posthuman Education by depicting the interrelation of psychological depth, ethical reflexivity, and technological attunement within a shared field of awareness. The model emphasizes that leadership emerges through conscious mediation among emotional, moral, and technological intelligences operating within hybrid human–machine–ecological systems. It also reflects evolutionary-cognitive insights into how human adaptive capacities interact with digital infrastructures, situating conscious leadership within broader processes of human–AI co-adaptation.

### 2.5 Philosophical and ethical grounding

The Conscious Leadership Model is grounded in a philosophical synthesis integrating phenomenology, posthuman ethics, and relational ontology. Phenomenology (Husserl, 1913; Merleau-Ponty, 1945/1962) informs the model’s emphasis on consciousness as lived and embodied experience rather than abstract cognition. From this perspective, awareness is not a detached mental function but an emergent process arising through situated engagement with the world. This understanding resonates with Varela et al.’s (1991) enactive approach, which conceptualizes cognition as embodied, relational, and co-constituted within dynamic systems.

Posthuman ethics (Braidotti, 2013; Ferrando, 2019) extends this orientation by situating moral responsibility within networks of human, technological, and ecological interdependence. Rather than framing leadership as unilateral control, this perspective emphasizes relational accountability and responsiveness within hybrid socio-technical environments. The inclusion of technological mediation within ethical reflection does not negate human responsibility; rather, it acknowledges that moral agency is increasingly enacted within infrastructures that shape perception, choice, and institutional outcomes. In this regard, Floridi’s (2014) concept of the “infosphere” provides a useful framework for understanding how digital systems participate in structuring moral environments.

The relational ontology underpinning the model positions consciousness as emerging through interaction rather than existing as an isolated substance. Within leadership studies, this ontological stance aligns with relational and complexity-oriented approaches that view leadership as processual, distributed, and co-created (Uhl-Bien, 2006; Day, 2014). By linking phenomenological insight with posthuman ethics, the present framework suggests that conscious leadership can be understood not only as a psychological capacity, but also as an ethical orientation enacted within complex socio-technological contexts.

In this sense, the model does not propose a replacement of existing leadership paradigms, but a deepening of their ontological foundations—foregrounding awareness as the medium through which relational, ethical, and technological dynamics are integrated in educational practice.

## 3 Methodological orientation: conceptual synthesis

This study adopts a conceptual synthesis methodology (Jaakkola, 2020; Torraco, 2016) to integrate and extend theoretical perspectives from posthuman philosophy, transpersonal psychology, ethics of technology, and educational leadership. Conceptual synthesis differs from empirical inquiry in that its primary aim is theory development rather than data generation. Recent methodological discussions have further clarified the standards of rigor expected in theory-building research within interdisciplinary fields. Scholars emphasize that high-quality conceptual articles must demonstrate explicit problematization of existing assumptions, systematic engagement with relevant literatures, and transparent articulation of their integrative logic (Jaakkola, 2020; Post et al., 2020; Cornelissen, 2017). In rapidly evolving domains such as AI-mediated education, conceptual development plays a critical role in clarifying ontological shifts and normative implications before stable empirical frameworks can be established. Accordingly, this study positions conceptual synthesis not as a preliminary or speculative exercise, but as a necessary mode of scholarly intervention responsive to emerging socio-technical transformations. The rigor of such work rests not on statistical validation but on systematic integration, conceptual coherence, and analytical transparency.

The development of the Conscious Leadership Model followed three iterative phases. First, a selective and purposive review of contemporary scholarship on AI-mediated education, posthuman theory, and relational leadership was conducted to identify recurring conceptual tensions concerning agency, consciousness, and technological mediation (Knox, 2023; Jandrić and Ford, 2020; Williamson, 2017). Second, foundational theoretical frameworks from phenomenology, transpersonal psychology, and enactive cognition were analyzed to clarify how consciousness has been conceptualized beyond individualist paradigms (Ferrer, 2002; Varela et al., 1991). Third, these strands were integrated through abductive reasoning to generate a coherent model linking psychological depth, ethical reflexivity, and technological attunement as interdependent dimensions of leadership awareness.

Throughout this process, conceptual integration was guided by four criteria of theoretical rigor (Jaakkola, 2020): internal consistency, explanatory scope, integrative capacity across disciplines, and relevance to contemporary educational challenges. Rather than aggregating perspectives, the synthesis sought to articulate relational patterns among them, identifying shared ontological assumptions and points of productive tension. The resulting model therefore represents a structured theoretical contribution grounded in established traditions while extending them into posthuman educational contexts.

Epistemologically, the study is informed by participatory and enactive paradigms of knowledge (Ferrer, 2002; Varela et al., 1991), which view understanding as emergent and relational. However, this orientation does not imply relativism; instead, it acknowledges that theory-building in complex socio-technical environments requires reflexive awareness of how conceptual frameworks shape interpretation. By explicitly articulating its philosophical commitments and analytical steps, the study aims to ensure transparency and scholarly accountability.

The contribution of this methodology lies in reframing conscious leadership as an ontological and ethical construct emerging at the intersection of human and algorithmic intelligences. By systematically integrating transpersonal depth, posthuman ethics, and leadership theory, the model advances discourse beyond functionalist or purely managerial interpretations of leadership (Jandrić and Ford, 2020; Knox, 2023). Conceptual synthesis, in this context, functions not as speculative abstraction but as disciplined theoretical development responsive to rapidly evolving educational realities.

## 4 Discussion: implications for educational leadership

The Conscious Leadership Model contributes to ongoing debates concerning the future of educational leadership in digitally mediated contexts. Rather than positioning itself in opposition to established leadership paradigms, the model extends relational, distributed, and complexity-oriented approaches by foregrounding consciousness as an analytic and ethical dimension of leadership practice (Uhl-Bien, 2006; Day, 2014). In environments increasingly shaped by artificial intelligence and data-driven governance, leadership unfolds within socio-technical systems that mediate judgment, accountability, and institutional culture. The model therefore invites reconsideration of how awareness operates within such systems.

From a professional development perspective, the framework highlights three interrelated domains of leadership formation: reflective self-awareness, ethical discernment, and critical engagement with technological infrastructures. These domains do not replace managerial competence; rather, they situate it within broader moral and relational contexts. As Knox (2023) suggests, ethical leadership in AI-mediated environments requires bridging systemic accountability with reflective presence. The present model builds on this insight by articulating how psychological depth, ethical reflexivity, and technological attunement may function as mutually reinforcing capacities in digitally complex institutions.

Institutionally, the framework aligns with systems and relational theories of leadership that conceptualize organizations as dynamic ecologies of meaning-making (Senge, 1990; Uhl-Bien, 2006). Within such ecologies, accountability may be understood not solely as performance measurement but as reflective responsiveness to human and technological interdependencies. By incorporating awareness of algorithmic mediation into leadership practice, educational institutions may be better positioned to navigate tensions between efficiency, equity, and ethical responsibility.

Recent research on public sector AI and algorithmic governance further underscores that leadership in data-driven institutions entails navigating complex accountability regimes shaped by automated decision systems (Wirtz et al., 2019; Valle-Cruz et al., 2020). Studies examining AI implementation in public administration highlight that algorithmic systems often redistribute discretion rather than eliminate it, requiring leaders to reinterpret procedural justice, transparency, and oversight within digitally mediated governance architectures. In educational contexts, this suggests that leadership development must address not only technological competence but also institutional capacities for auditing, explainability, and participatory oversight. Such considerations align with emerging international policy frameworks that emphasize human-centered AI governance in public services (OECD, 2021; UNESCO, 2023), reinforcing the model’s claim that conscious leadership functions as an interpretive and ethical interface within socio-technical systems.

### 4.1 Policy-level considerations and research directions

At the policy level, the model suggests that leadership preparation programmes could benefit from integrating ethical literacy and critical digital awareness alongside technical competencies. Rather than prescribing specific reforms, this perspective highlights the potential value of incorporating reflective methodologies into leadership education—particularly in contexts where AI systems influence institutional decision-making.

Because the Conscious Leadership Model is conceptual, its practical implications warrant empirical exploration. Future research might investigate how leaders experience ethical tension in AI-mediated environments, how reflective practices shape decision-making under algorithmic governance, or how technological attunement manifests across cultural contexts. Comparative and qualitative studies could further clarify how consciousness, understood as relational awareness, is enacted in diverse educational systems.

### 4.2 Implications for leadership development

Translating the model into practice involves reconsidering how leadership competence is defined and assessed. Contemporary leadership development often emphasizes strategic planning, data literacy, and performance management. The present framework suggests complementing these competencies with structured opportunities for reflective dialog, ethical deliberation, and critical engagement with digital infrastructures.

Practices such as dialogical inquiry, peer reflection, and interdisciplinary collaboration between educators and technologists may support leaders in developing sensitivity to how algorithmic systems shape institutional life. When combined with emotional intelligence and systems awareness (Goleman, 1998; Senge, 1990), such practices may enhance leaders’ capacity to navigate complexity without reducing it to technical problem-solving alone.

Importantly, awareness cannot be operationalized as a metric in the same manner as performance indicators. Rather, it emerges through ongoing reflective engagement within institutional cultures. Leadership development initiatives that cultivate relational competence and technological discernment may therefore contribute to more ethically responsive educational systems.

### 4.3 Evolutionary mismatch and algorithmic authority

From an evolutionary-cognitive perspective, the rise of algorithmic governance introduces potential tensions between evolved social heuristics and abstract forms of digital authority. Human trust mechanisms developed in small-group contexts characterized by embodied cues—tone, gaze, emotional reciprocity, and shared presence. Algorithmic decision systems, by contrast, often operate through impersonal data processes lacking these relational signals (Waytz et al., 2010).

This mismatch may generate both over-trust and distrust toward algorithmic outputs, depending on context. Within educational leadership, conscious mediation becomes relevant insofar as leaders interpret and contextualize algorithmic recommendations rather than deferring to them uncritically. In this way, the model situates technological authority within broader human capacities for ethical reflection and social judgment, linking evolutionary inheritance with posthuman conditions.

In this context, the algorithmic self must navigate the tension between evolved social intuition and the logic of automated systems. This requires moving beyond a mere instrumental use of technology toward a “pedagogy for a world with AI,” where leadership involves the continuous monitoring and critical interpretation of algorithmic agency (Bearman and Ajjawi, 2023).

## 5 Conclusion: toward a posthuman ethics of educational awareness

This study has introduced the Conscious Leadership Model as a theoretical framework for examining educational leadership within AI-mediated and posthuman contexts. By integrating psychological depth, ethical reflexivity, and technological attunement, the model extends relational and complexity-oriented leadership theories into domains increasingly shaped by algorithmic infrastructures. Rather than displacing established paradigms, it reframes leadership as a relational process unfolding within socio-technical systems where human judgment and technological mediation intersect.

At its core, the framework advances the argument that digital transformation in education is not solely a technical challenge but also an ontological and ethical one. As artificial intelligence becomes embedded in institutional decision-making, questions concerning awareness, agency, and responsibility acquire renewed urgency. The model therefore situates consciousness not as an abstract ideal, but as an analytic and practical orientation through which leaders interpret and mediate algorithmic authority.

In this study, consciousness is employed as a conceptual-analytic category rather than as a directly measurable psychological variable. It designates a mode of reflective awareness and relational attunement that structures how agency is enacted within socio-technical environments. The model is therefore intended as a heuristic and normative framework that invites further empirical elaboration, rather than as a finalized psychological theory.

This perspective suggests that leadership preparation and educational governance may benefit from incorporating reflective, ethical, and technologically critical dimensions alongside managerial competencies. However, such integration does not entail abandoning efficiency or accountability; rather, it involves situating them within broader relational and moral contexts. In this sense, the model contributes to a growing body of scholarship seeking to align educational innovation with ethical responsibility (Knox, 2023; Jandrić and Ford, 2020).

Recent international policy and research agendas increasingly emphasize that the integration of artificial intelligence into public education systems requires new forms of human-centered governance capable of ensuring transparency, fairness, and meaningful oversight (OECD, 2021; UNESCO, 2023; European Parliament and Council of the European Union, 2024). These developments suggest that educational leadership will progressively operate within regulatory environments that formalize requirements for explainability, risk assessment, and accountability in algorithmic systems. Within such contexts, conscious leadership may function not only as an ethical stance but as an institutional necessity—supporting leaders in mediating between technical infrastructures, regulatory frameworks, and pedagogical values.

The concept of the algorithmic self further extends this contribution by proposing a relational understanding of consciousness emerging at the intersection of evolved social cognition and digital mediation. Viewed through both posthuman and evolutionary lenses, this construct highlights how human awareness adapts within hybrid human–machine ecologies. Conscious leadership, therefore, may be interpreted not only as an ethical orientation but also as a cognitive adaptation to increasingly complex socio-technical environments.

Because this study is conceptual, future research is needed to examine how these theoretical propositions manifest in practice. Empirical and phenomenological investigations could explore how leaders experience algorithmic mediation in decision-making processes, how ethical reflexivity is enacted under conditions of digital governance, and how technological attunement develops across institutional contexts. Narrative, comparative, and interdisciplinary approaches may further illuminate the lived dimensions of posthuman educational leadership.

Ultimately, this paper proposes that educational leadership in the algorithmic age can be understood as an ongoing negotiation between human depth and technological complexity. By foregrounding awareness as a mediating capacity within hybrid systems, the Conscious Leadership Model offers a theoretically grounded contribution to discussions of ethics, agency, and responsibility in contemporary education.

As such, the Conscious Leadership Model should be read as a generative framework designed to stimulate interdisciplinary dialog and guide future inquiry into the ethical and cognitive dimensions of AI-mediated leadership.
